# Maternal multimorbidity - experiences of women seeking asylum during pregnancy and after childbirth: a qualitative study

**DOI:** 10.1186/s12884-023-06054-x

**Published:** 2023-11-13

**Authors:** Anna Rowe, Minakshi Bhardwaj, Mary McCauley

**Affiliations:** 1 Liverpool Women’s NHS Foundation Trust, Crown Street, Liverpool, L8 7SS United Kingdom; 2 Department of International Public Health, Liverpool School of Tropical Medicine, Pembroke Place, Liverpool, United Kingdom; 3 College of Nursing, Midwifery and Health Care, University of West London, London, United Kingdom

**Keywords:** Maternal multimorbidity, Asylum seeker, Pregnancy, Antenatal care, Maternal mental health, Social wellbeing

## Abstract

**Background:**

Many women seeking asylum during pregnancy and after childbirth have ill-health but detection and assessment of all physical, psychological, and social health needs (maternal multimorbidity) are often difficult as part of routine maternity care. Healthcare providers are key for the early identification and management of vulnerable pregnant women who have additional physical, psychological, and social health needs. We sought to explore the impact of the asylum-seeking process, understanding of wellbeing, expressed health needs (in terms of maternal multimorbidity), and the experiences of maternity care of women seeking asylum during pregnancy and after childbirth in Liverpool, United Kingdom. Enabling factors and barriers to access woman-centred care were also explored.

**Methods:**

Key informant interviews (*n* = 10) and one focus group discussion (*n* = 4) were conducted with women attending a non-profit charitable pregnancy support group. Transcribed interviews were coded by topic and then grouped into categories. Thematic framework analysis was undertaken to identify emerging themes.

**Results:**

The asylum-seeking process negatively impacted women making them feel anxious and depressed with little control or choice over their future. Women reported feeling stressed regarding poor standard of accommodation, low income, dispersal and the uncertainty of their asylum application outcome. Wellbeing during pregnancy and after childbirth was understood to be multifactorial and women understood that their physical health needs were interlinked and negatively impacted by complex psychological and social factors. Women reported that their expectations of maternity services were often exceeded, but information giving, and the use of language interpreters needed to be improved. Women expressed the need for more psychological and social support throughout pregnancy and after childbirth.

**Conclusions:**

A multidisciplinary team, with links and effective referral pathways to maternal mental health and social services, are necessary for women seeking asylum, to ensure a more integrated, comprehensive assessment of maternal multimorbidity and to provide maternity care in a way that meets all health needs.

**Supplementary Information:**

The online version contains supplementary material available at 10.1186/s12884-023-06054-x.

## Background

The health needs of women seeking asylum, during pregnancy and after childbirth include complex physical, psychological and social comorbidities and is an international public health concern [1–6]. The Sustainable Development Goal 3 is to improve the health and wellbeing for all at all ages by 2030, and the Global Strategy for Women’s, Children’s and Adolescent’s Health emphasises that all women have the right to, and should obtain, the highest attainable standard of health [7, 8]. Many women seeking asylum during pregnancy and after childbirth experience maternal multimorbidity and commonly this ill-health remains undetected, untreated or is recognised late [1, 2]. Maternal morbidity has been defined as ‘any health condition attributed to and/or aggravated by pregnancy and childbirth that has a negative impact on women’s well-being’ [9]. This has been argued to constitute physical, psychological and social conditions [1]. In this study the term ‘maternal multimorbidity’ is used to describe the negative impact of more than one physical, psychological or social condition on wellbeing during pregnancy and after childbirth. It is important to understand the experiences of and address the multiple complex health needs of women seeking asylum during pregnancy and after childbirth, in order to decrease morbidity and mortality [1–4]. Moreover, studies that distinguish women who are seeking asylum from those who have refugee status or have migrated to the host country for other reasons, are sparce.

Numerous studies have shown that refugee and migrant women, including those seeking asylum, during and after pregnancy are more vulnerable with higher rates of maternal and newborn morbidity and mortality compared to local populations of the host country. Studies have found that this group of women, are seven times more likely to develop complications and three times more likely to die during labour [10–13]. In the latest MBRRACE report 495 women died during or up to one year after the end of pregnancy in the UK (between 2017–2019), and 8% (*n* = 40) had severe and multiple disadvantages, of which refugee or asylum seeker status, and arrival in the United Kingdom (UK) within the five years are significant risk factors [10]. In addition to increased rates of maternal mortality, rates of multimorbidity, especially psychological ill-health, are high for women seeking asylum during pregnancy and after childbirth because of limited healthcare provision in their home country and/or during the migration journey [14]. An estimated 40% of refugees are affected by post-traumatic stress (PTSD), depression- and anxiety disorders and women seeking asylum during pregnancy and after childbirth are at greatest risk [15]. Furthermore, women seeking asylum have a higher risk of Caesarean birth and adverse perinatal outcomes, including pre-term delivery, low birth weight, congenital malformations and newborn morbidity and mortality [16–19].

Many high-income countries have excellent services and resources available for routine comprehensive and woman-centred antenatal and postnatal care provided by motivated and highly skilled healthcare providers [20–23]. When a woman seeking asylum is pregnant and engages with the health system, there is a ‘window of opportunity’ for healthcare providers to provide best quality care including a comprehensive assessment and management of maternal multimorbidity, alongside complex social factors [24–27]. However, significant challenges and barriers currently remain for women seeking asylum including social isolation, language barriers, availability of interpretation services, and lack of specialist multidisciplinary services linked with maternal mental health and social support services [11–19, 27]. This study sought to explore the impact of the asylum-seeking process, understanding of wellbeing, expressed health needs, and the experiences of maternity care of women seeking asylum during pregnancy and after childbirth in Liverpool, United Kingdom. Enabling factors and barriers to access woman-centred care were also explored.

## Methods

### Study design and setting

In-depth key informant interviews (*n* = 10) and one focus group discussion (*n* = 4) were conducted with women attending a non-profit charitable support group: the Merseyside Refugee and Asylum Seekers Pre and Post Natal Support Group (MRANG) (now re-named the Refugee Women Connect) in Liverpool, UK in 2017 [28].

The use of one-to-one interviews enabled the exploration of sensitive issues due to providing a more private environment, necessary to enable participant safety and trust [29].

A focus group discussion (FGD) was chosen in order to triangulate data and because FGDs can be used to both examine what people think and why they think a certain way and therefore would be important in revealing the participants’ hopes and expectations of care during pregnancy and after childbirth [30]. Importantly, this method allows the researcher to tap into interpersonal communication, highlighting cultural values and shared understanding.

All interviews, including the FGD took place in the most convenient place for the woman, either in their home or in a private room at the MRANG drop-in centre to ensure privacy.

### Participants

Women seeking asylum were included if they were currently pregnant or had given birth in the UK within the previous two years. Snowballing and opportunistic sampling techniques were employed to identify the participants [31]. Participants were chosen purposively, based on their ability to speak and understand English in order to reflect on their experiences, and were recruited sequentially until data saturation was met. Data saturation is a qualitative research principle that describes the point in the data collection and analysis stage where ‘additional data does not lead to any new emergent themes’ and therefore recruitment of further participants is no longer necessary to reach conclusions [32].

### Topic guide

A topic guide was developed and piloted at the study site. The topic guide was a flexible tool that enabled the interviewer to capture women’s’ responses as well as acting as a cue to probe further to understand the participants’ experiences (Supplementary File 1). In addition to sociodemographic questions, the topic guide included four main subject areas: (1) the impact of the asylum-seeking process on women’s health and wellbeing; (2) women’s understanding of health and wellbeing during pregnancy and after childbirth; (3) women’s specific health needs; and (4) the lived experiences women seeking maternity care. Recommendations of how health and social care providers can improve maternity care were also explored.

### Data collection

Key informant interviews and the focus group discussions were conducted face-to-face in English, lasted on average 65 min, were recorded on a digital recording device, and transcribed on completion. Anonymity and confidentiality of data was emphasised to reassure participants’ confidence in providing honest answers.

### Analysis

The key informant interviews and focus group discussion were transcribed verbatim. Data was entered into NVivo by electronically highlighting excerpts from the transcripts and placing them under the relevant code. Framework analysis was used to discover similarities between the experiences from the narratives of women seeking asylum [33]. A framework was created based on recurring themes that arose during the familiarisation stage. The same analytical approach was used to analyse the key informant interviews and focus group discussion, with a deductive framework based on the topic guide. The first author (AR) independently coded all transcripts. A subsample of transcripts was independently coded also (MB, MMC) and the identified codes were grouped into categories and reviewed by all three researchers (AR, MB, MMC) to ensure consistency. This enabled the first extraction of data. Key themes were discussed and checked by all researchers together to reach consensus. We used the Standards for Reporting Qualitative Research guidelines in reporting the analysis [34].

### Reflexivity

To facilitate transparency and reflexivity, a reflective diary was kept throughout the research process to evaluate and address researcher bias. Participant checking took place after the first interview whereby the transcript was checked with the key informant to ensure meaning was not lost through misinterpretation to increase internal validity of data. For subsequent interviews, participant checking took place during interviews if meaning was not clear. Initial interpretation of the data was undertaken by the first (AR) and second author (MB) and data was then further analysed by a third author (MMC). All authors have different professional and cultural backgrounds which helped to mitigate personal biases and avoid the pitfalls of only one viewpoint.

### Ethics

Ethical approval was granted by the Research Ethics Committee at the Liverpool School of Tropical Medicine, UK. Written informed consent was obtained from all participants of the study. All methods were carried out in accordance with relevant guidelines and regulations in the declaration of Helsinki.

Research with people seeking asylum raises some of the most complicated ethical issues and thus requires rigorous background research to improve sensitivity towards cultural differences and traumatic life events in addition to the building of trust between researcher and participant [35–38]. Guidance was sought throughout the research process from MRANG staff who have built trust with women seeking asylum and consequently have greater cultural awareness.

Maintaining confidentiality is of utmost importance to protect the identity of the women seeking asylum and was ensured by anonymising all data collected from participants and in transcripts (physical and electronic). All electronic data was anonymised and stored on a password-protected computer. All women were assured of the measures to maintain confidentiality and any questions they had about the process were answered before consent was gained.

Due to the nature of interview questions, steps were taken to ensure women felt safe and had full control over stopping the interview at any point if they felt it was too difficult to continue. Psychological support by qualified professionals, accessed through MRANG was offered to women participating.

## Results

### Participants’ characteristics

Fourteen women participated in the study (five during pregnancy, nine after childbirth). The age of participants ranged between 24 and 47 years, with the median age of 33 years old. Participants were from seven different countries: Nigeria (5); Albania (2); Egypt (2); Pakistan (2); Iran (1), Georgia (1), and Namibia (1).

### Emerging themes

The main emerging themes are presented below with illustrative quotes provided in Tables 1, 2,3, 4 and 5. When describing the themes, we use the term ‘women’, not to describe all women in general and their views but highlight that these results are specific only to women who participated in this study.

#### Theme 1. The impact of the asylum-seeking process on women’s wellbeing in pregnancy and after childbirth

Women described the negative impact of stress and anxiety related to the asylum-seeking process on their physical, psychological and social wellbeing. One woman expressed that the most important priority in pregnancy was ‘to be calm and not to… be [an] asylum [seeker] or refugee’ (Table 1, Q1). Women reported that the uncertainty of the asylum application outcome negatively impacted their sense of control and future safety, resulting in anxiety and stress (Table 1, Q2-4). These feelings were exacerbated by having poor knowledge of essential systems in the UK, particularly how to apply to the National Asylum Support Service (NASS), and feelings of frustration that they did not have the freedom of choices or resources to change their situation without the right to work (Table 1, Q 5–7).

**Table 1:** The impact of the asylum-seeking process on women’s wellbeing in pregnancy and after childbirth

Q1	*‘The most important is to be calm and to not to have, not to be asylum or refugee’* (KII 1)
Q2	*'I was so scared, I was under the pressure of giving interview, I do not know what will happen; what if they refuse; what if they straightaway refuse me?'* (KII 10)
Q3	*‘I am having two kids already, and they are still young, and I am still pregnant and I am still seeking for an asylum. you know- the stress from Home Office, everything, thinking. I am not just- during this pregnancy, I am not happy at all- I am not happy’* (KII 7)
Q4	‘*Already I can't have relaxation for this time because err I should go follow solicitor and I…can't control myself also I have the stress about my situation in the Home Office’* (KII 2)
Q5	*‘I didn’t know even how to apply for the NASS (National Asylum Support Service) support. And I did on my own and they refused. Because I didn’t know how to do, how the things work’* (KII 6)
Q6	*‘I come from my country to be safe. Why? You don’t ask it, Home Office don’t ask himself why? Because I have a problem really and I need to be safe. And I need to live here in the good situation, not the same situation. If I know I go through all of these things, same here, I live here with all of this stress. The same stress, when I'm far from… from here to here to here, to here to here, but I have money, in my country, because I work'* (KII 9)
Q7	*‘For refugees for people from other country where there is bombing or war or something, they feel comfortable here because they are safe and the only problems they had was the safeness, but for me now I feel safe but I have more problems. I'm sitting all the time at home, I have no right to work, no one needs my knowledge, no one needs, because I don't have NASS support I can't study, I can't go in a college or you know I'm sitting all the time at home, I can't do anything. Who cares I have finished Law, Masters of Law or anything. For Home Office, for England, I am just a person who lives. If I did not have children I would get crazy because I do not have the right… I have something to do. If I did not have them I would just sit at home. It's terrible.’* (KII 1)

#### Theme 2. Women’s understanding of health and wellbeing during pregnancy and after childbirth

In this study, women understood wellbeing during pregnancy and after childbirth to be multifactorial, including physical, psychological and social factors (Table 2, Q1-2). Women recognised that their wellbeing during pregnancy was important as it influenced their ability to take care of themselves and their baby (Table 2, Q3–4). Support from a partner or family member reduced feelings of stress and anxiety (Table 2, Q5-6). A ‘good’ pregnancy was regarded as being free from stress, having a healthy baby, feeling strong and healthy, feeling prepared for birth, and having support from partners and healthcare providers (Table 2, Q7-12). The majority of women described a ‘good’ birth as a normal vaginal delivery with no complications. Having choice in the type of delivery and effective pain relief was important (Table 2, Q13-16).

**Table 2:** Women’s understanding of health and wellbeing during pregnancy and after childbirth

**Wellbeing in pregnancy and after birth**	
Q1	*‘It’s about dealing with pregnant women and their wellbeing; their physical and emotional well-being.’* (KII 8)
Q2	*‘My social factors, physical factors…I think they are all the same. They are all the same’* (KII 7)
Q3	*‘I think it’s not being stressful and to be able to look after the baby.’* (KII 5)
Q4	*‘It’s very important because if you're not well you can't look after the baby and you can't look after your other children as well so being well is very important so you can continue that’* (KII 3)
Q5	*‘When you are seeing your partner with you, you see people around you, you are happy, you understand, you are sharing, you are happy, you are whole, you understand?’* (KII 7)
Q6	*‘You see so, when I have been pregnant, you see your partner beside you that’s good. But if someone is pregnant does not see your partner, do not see anybody such that’s hard’* (KII 4)
**A good pregnancy**	
Q7	*‘Good pregnancy need to be in a rest, in a good place, do not speak anything that makes you stressed’.* (KII 9)
Q8	*‘Where your baby is health, I think is ok…there is no complication, as the close date, both of you are life, think that should be ok’*.(KII 3)
Q9	*‘My health. You have to know if you are well.’* (KII 8)
Q10	*‘So, if you just plan to get pregnant, and you should both agree, your circumstances are good. Then if you get pregnant, then it’s going to make a difference, I think. You going to have a healthy pregnancy and healthy child’.* (KII 6)
Q11	*‘ If you are pregnant and you are depressed, that’s not a good pregnancy’.* (KII 7)
Q12	*‘Yeah, for me you should have really good support. For the people like me, they do not know anything. There should have somebody to guide them, to tell them the things. Like the volunteers who work here, I have not seen in London. So, midwife or whatever, they should guide them properly in a proper way. So, it’s going to be such a beneficial for the healthy pregnancy’.* (KII 6)
**A good birth**	
Q13	*‘I want to try natural born baby, it's very better and very good for healthy, for me and my baby also’* (KII 2)
Q14	*‘I think the best birth is the, vaginal birth is the best. Yeah. Because you go through the pain and after having the baby, then after having the baby you are free. Than having the baby, say, after having my baby, you still go through pains. so, it’s better. So, I think it’s better.’* (KII 7)
Q15	*‘Err yeah with the help of the erm… what's it called?… helpful when you are err, Gas and air yeah, yeah this is ok’* (KII 3)
Q16	*‘It’s a good thing that you have a choice (of delivery) so that you can get rid of pain. Even in caesarean, after giving birth you still have pains. I am still having the pain or you go through the pains and then after having the baby you become okay. So, it’s an easy choice. So, it’s good to make a choice.’* (KII 4)

#### Theme 3. Women’s specific health needs: cultural and language challenges

Women described multiple cultural challenges such as lack of maternity knowledge, contrasting clinical practices between home and host country; lack of familial support; and limited access to familiar food. One woman explained that she had no prior experience or knowledge of pregnancy and felt lost and overwhelmed, feelings that were exacerbated by the absence of her family, especially female members, who would normally provide additional support during pregnancy and after childbirth (Table 3, Q1). Some women described cultural misunderstandings and differences in caring for babies such as umbilical cord care and how this caused distrust in relationship with healthcare professionals (Table 3, Q2). Women highlighted the challenges associated with not being able to find food that they were used to eating and having to cook unfamiliar foods, which caused hunger during their pregnancies (Table 3, Q3–4).

**Table 3:** Women’s specific health needs: cultural and language challenges

**Cultural challenges**	
Q1	*‘Usually unmarried girls do not know much about like, how to do things like that… So, when I came here, I did not know anything. Like when you get pregnant, this happens, that happens. This is no good. And I was just going to the doctor again. I just carried a lot of stress because not knowing anything… when the lady, when she gets pregnant [in Pakistan], it’s the family who looks after her*.’ (KII 10)
Q2	*‘For me, when I gave birth in my home town, you have to use a bandage to tie it around the navel so that it come out nice. Here you have to leave it natural so that it's clean, so it was very strange for me and all the time I was worried. And one day, I bandaged it for in my country you have to cover it so that no breeze will get to it and the midwife came and she was a bit, she had that look that she saw I had done that and …but She didn't try to understand that it was done to me, it was done to everyone coming from my country. So, after that I had to take the bandage off, she started coming without even telling me to see if I was still doing it. And… I stopped but I found it very difficult because every day I see the clip, I got scared and I started feeling, what’s happening?’* (FGD P2)
Q3	*‘I could not eat everything, especially I was not used to English food taste, so I could not even cook because I was in a hotel. So first two months I can say I was always hungry just cucumber and tomatoes.’* (KII 1)
Q4	*‘We spent 4 days over there and it was very cold, and we could not get any proper food, for I was pregnant and I was sick quite a lot. And we could not cook our food over there.’ (KII 10)*
**Language and information giving**	
Q5	*‘Finding an interpreter was not easy, because it’s on the language, and the Albanian is not always, they don’t always can have the interpreter, and sometimes, the appointment has to be cancelled or rearranged, so, even with GP and in hospital, so the language is’.* (FGD P1)
Q6	*When I had my first baby, it was very difficult, you know, very, very difficult. And I did not know how to say even a word, because I did not know no English at all. And because of pregnancy, because the labour was very difficult, and they were even saying to put me, you know, on C-section, but I was a… because I do not speak English, they did not know what to do, because I had to sign some consent like things like that. And it was very shocking. I was just crying, because when you do not understand anything.* (FGD P3)
Q7	*‘I do not know that but my doula told me that in Women’s’ hospital, they do all kind of classes…how you want to give birth. The births and…even then I had the C-section. But it was stressful like, it was in hospital anyway, and it was my doula who gave me the information because I did not know.’* (FGD P1)

Women discussed the need for accessible and appropriate information giving, effective communication, and the additional support of an interpreter to help them prepare for pregnancy and childbirth. The overriding barrier to effective information giving was language proficiency. Even though women in this study spoke conversational English, many explained that language barriers still existed, resulting in negative experiences including cancelled or delayed appointments (Table 3, Q5). Women reported that they would appreciate the use of an interpreter for all appointments to enable greater understanding and enable them to express their concerns more clearly. One woman reflected on the trauma of going through an emergency Caesarean section when she did not fully understand what was happening due to language barriers (Table 3, Q6). Language barriers also meant that women were told they could not attend antenatal classes as there was no interpreter provided and therefore, they felt unable to access useful information about pregnancy and childbirth in that setting. The use of doulas, even if they did not speak the same language was expressed as a helpful way to overcome some of these barriers by providing emotional support and accompaniment to appointments (Table 3, Q7).

#### Theme 4. The experiences of women seeking maternity care in terms of maternal multimorbidity

##### Physical morbidity

Many women reported feeling well supported by healthcare providers and felt that their physical health needs were often met. Positive healthcare experiences included appointments in hospitals with doctors and regular home visits from midwives and health visitors helping women to feel cared for and reassured (Table 4, Q1-4). However, a small number of women felt unsupported and described negative experiences including not knowing how to access healthcare services appropriately, delays in accessing community and specialist care, language barriers; and miscommunication regarding health concerns (Table 4, Q5-6). One woman described feeling discriminated against when her health concerns about her low platelet count were dismissed by healthcare providers (Table 4, Q7). This resulted in severe anxiety about dying during her elective caesarean section which led to a traumatic birthing experience. She believed that concerns about her low platelet count were not addressed adequately because she was not English, which acted as a barrier to positive interaction with healthcare providers (Table 4, Q7).

**Table 4:** Lived experiences of women seeking maternity care in terms of maternal multimorbidity

**Physical Morbidity**	
Q1	*‘Yeah here, about baby I haven't any worry err for example for sonography, for any test, for NHS, everything about NHS. It's very good after my country.’* (KII 2)
Q2	*‘I go to women hospital only. Before I pregnant, I go to another hospital. But women hospital is very good, very good. I am feeling good, because I see him care about my pregnant, care about me. I see that.’* (KII 9)
Q3	*‘This midwife just came to look see me.. she came yesterday, she came today and she said she's coming again on Tuesday, you understand? The care is there… just check after me, even when I told them I am fine they keep coming to check like this. Those are good things that are encouraging and still hope to carry on.’* (KII 3)
Q4	*‘It’s good. yes. It’s good. They (healthcare professionals) always nice to me, talk to me. Even the midwives, they always double check. That’s so nice. They were so nice.’* (KII 4)
Q5	*‘More than two weeks or three weeks I tried to get to doctor (GP) but I could not see. They need to fill in some forms.’* (KII 1)
Q6	*‘The only thing he said that you can go to A&E, if you have ever feel like that (have a panic attack). And when you go to A&E, the first thing is you have to see a nurse. So, have to explain to the nurse, and they will keep about you 2–3 h waiting just that someone can talk to you. Within that time, just the waiting, because you want to see someone, just the waiting, its like it gets you high again because you feel like you are being abandoned. So, its just worsen the situation, when you wait in A&E.’* (KII 8)
Q7	*‘I wanted to see (the Haematologist) before Caesarean because I did not want to die! the Anaesthetist told me he’s too late now…. Sometimes I was thinking that if I was English, it did not happen … I did not know I would sleep whilst Caesarean or not because they explained it was dependent on my platelets… So all my pregnancy I don’t know why it happened, but no one tried to raise my platelets. I don’t know. And even after Caesarean I wanted to find out what’s happened with my platelets. Even now I don’t know.’* (KII 1)
**Psychological Morbidity**	
Q8	*‘They say if you are stressed you might have a miscarriage. And most of the time, being an asylum seeker is stressful.’* (KI 8)
Q9	*‘You need to be relaxed, doctor tells you but you need to be relax for your baby may be not good because you are in stress a lot of the time.’* (KII 9)
Q10	*‘Once when I was in the detention centre, I tried to kill myself with tablet.’* (KII 2)
Q11	*‘Yeah… because during the pregnancy, I tried to harm myself. I want to do bad things to myself, to kill myself. and so that. And there was nobody to support me.’* (KII 4)
Q12	*‘When I was pregnant, I used to go when I had appointment. But nobody used to discuss with me about my mental health and anything. They just used to examine, the basic examinations for pregnant lady. They used to do the weight and I really do not remember what they do. But they do not talk about my mental health, they never discussed whichever problems I had.’* (KII 6)
Q13	*‘One of the issues with the NHS is that they don’t give enough information about facilities available to pregnant women…For instance, like I said earlier, obviously during your pregnancy, you have an emotional breakdown, or you become stressed, and there are not enough numbers to call, for you to get the nurse or doctors. And sometimes, when you call your GP to say that I am feeling unwell and ask for an appointment, it takes about, you get it in 2 weeks, 3 weeks, and by the time you go in, everything is like a bit normal’.* (FGD 1)
**Social Morbidity**	
**Financial**	
Q14	*(Answering about her daily allowance) ‘It’s nothing. That’s why I always come to foodbank. They will give us cereal, food, milk, pasta. They say they are going to cook. But that £5 is nothing!’.* (KII 4)
Q15	*‘It [low income] has affected me looking after myself. I prefer buying for her, I prefer buying her wipes, her pampers, everything she needs, the essentials she need before I can buy things for me. And by the time I finish buying her things, I am left with little or nothing at all. So its kind of, its really difficult.’* (KII 8)
**Accommodation**	
Q16	*‘We did not have any proper room even. We have a small room…especially the area where we used to live there were mouse in the house. And in our room as well.’* (KII 6)
Q17	*‘I lived on the second floor and I fall on the stair, you know what happens in this house, the stairs are so high and it's so hard…And sometimes for cooking, for example I want to cook lunch, I should go in the up and down [the stairs], five times, three and six times, it’s very hard.’* (KII 2)
Q18	*‘Then we had after a few weeks err another woman with child was taken [to] this house where we lived so it was shared house and it was most difficult part of my pregnancy.’* (KII 1)
**Dispersal**	
Q19	*‘I have some organization. They come and meet me at home. They would stay with me. They would take me out for coffee… They would stay with me, we go out together, talk together but here I did not see anybody. They are only coming from London. They only coming to say hello to me, see how am I feel. At least they were coming. But here nobody come. I miss everybody.’* (KII 4)
Q20	*‘But it was a very hard time. I could not eat anything like. And we have to travel a lot and then we were sent to Liverpool. It was a long distance from London to Liverpool. It was a little hard journey over there as well. And then we were sent to Liverpool. Over there we stayed there for a week or two and it was again, you know, a lot of stress, like, you do not know…. And they said to me like you have to start your reporting from there. Finding ways, we did not have any car or stuff like that, you have to go by yourself. Going by bus, walking too much, weather is cold because it was raining in December’* (KII 10)
Q21	*‘During the process (of dispersal), I missed most of my appointments, my antenatal, post-natal, all my appointments… And I do not know. After I spent about two months not seeing any medical personnel or so. And when they finally saw me, my blood pressure was up and my, and I have blood clot and I had to be admitted for two days because, I was going and the nurse said it’s better that we saw you today because it’s not safe for you to be like that. And I said I spend two months not seeing any medical personnel… And the, my iron was very low as well. And I did not know anything about that and she said its whole affected me very badly’.* (KII 8)
Q22	*'Say, on Monday they call you and say tomorrow or day after tomorrow, you are going to be moved. So you do not have time. So that’s why when I move now, I do not even unpack my things, I just leave them as it is. I do not know when they are going to say, you are going to be moved again'. (KII 8)*

##### Psychological morbidity

Many women reported feelings of depression and anxiety during pregnancy and after childbirth and understood that psychological stress was detrimental to the pregnancy experience. Some women feared that feeling stressed would cause adverse outcomes for their baby, including miscarriage (Table 4, Q8-9). Some women described self-harming, having suicidal ideation and attempted suicide because of their circumstances (Table 4, Q10-11). One woman discussed how it was very difficult to access psychological support as her mental health was not screened at her antenatal appointments (Table 4, Q12). Another woman felt that she was not given enough information about who she could call for an acute mental health crisis (Table 4, Q13).

##### Social morbidity

Most women felt the income they received did not fulfil basic needs and resulted in dependency on charitable donations to survive and some women reported having to choose to feed and clothe their baby over themselves (Table 4, Q14-15). Many women were discontented with their living situation and reported poor housing conditions, lack of space, lack of choice and sharing a house with strangers particularly challenging (Table 4, Q16–17). Women reported that their housing needs were often dismissed, and sometimes felt unsupported and discriminated against. Some women were told that their housing expectations were too high. One woman described sharing a house as the ‘most difficult part’ of her pregnancy, which she attributed to a clash of cultures and behaviours from strangers sharing the accommodation (Table 3, Q18). Not having the ability to change her situation exacerbated this negative impact on her wellbeing. Dispersal, whereby asylum seekers are relocated away from one region of the UK to another part of the country, was described by several women to cause distress. For many, dispersal cut off invaluable support systems, caused women to travel for long periods whilst pregnant and aggravated the asylum-seeking process by making reporting to the Home office more difficult (Table 4, Q19-20). Another woman recalled how she missed many of her antenatal appointments as she was relocated three times during her pregnancy. Disruption to her continuity of care led to the late diagnosis of several significant medical conditions including hypertension, iron deficiency anaemia and deep vein thrombosis (Table 4, Q21). The uncertainty associated with dispersal delayed resettlement and resulted in one woman reporting that she was living out of her suitcase in case she had to move unexpectedly (Table 4, Q22).

#### Theme 5. Support from health and social care provider systems and charitable organisations

Women described positive and negative turning points in their asylum-seeking journey relating to the level of support they received from key services. Descriptions of support from healthcare providers that improved wellbeing during pregnancy and childbirth included: a non-judgemental, understanding and respectful attitude; consistent and reliable advice; healthcare providers who were encouraging, accessible, and sought to understand concerns. Receiving this support from healthcare providers and social workers encouraged positive coping mechanisms and behaviours that further facilitated women’s health and wellbeing during pregnancy and after childbirth. Women who received psychological support described that this helped them to overcome despair at their situation and focus on being mentally strong for their baby (Table 5, Q1–2). Charitable organisations such as MRANG were reported to be essential to fulfil the unmet needs of women. In addition to MRANG, local faith-based organisations and food banks provided food and/or financial aid when women did not have enough money to support their basic needs (Table 5, Q3). Many of the women discussed the pain they felt not having a partner, family or friends in the same country to support them during and after pregnancy, especially for emotional support. They explained that connecting with charitable organisations such as MRANG reduced the negative impact of isolation as they were able to make friends and access help that addressed their needs. MRANG promoted the health and wellbeing of women seeking asylum during and after pregnancy by providing a safe place to meet new people, emotional support from trusted professionals, information giving, signposting, and for companions to attend medical appointments (Table 5, Q4–7).

**Table 5:** Support from health and social care provider systems and charitable organisations

**Support from health and social care services**	
Q1	*‘The counselling was helpful, very, very helpful because the woman (midwife) keep telling me that you need to be strong, you need to focus on children, be strong for the children and the pregnancy as well so at least the counselling was really, really good’.* (KII 3)
Q2	*‘The social worker helped me. She called on behalf of me to the migrant help, to the Home Office and everything. She helped me really. And thanks God. We got the asylum support and they moved us to the Liverpool and Children centre, they helped us a lot. So, after having support from everywhere, I felt like very confident. Before, I felt that I am really alone. I do not have anybody, I do not have any support, I do not have anybody. But when they start supporting me, everybody was coming and visiting us-people from Children centre, the health visitor, the social worker they were looking after (baby X) and me and everything’.* (KII 6)
**Support from charitable organisations**	
Q3	*‘When I was pregnant and I was not able to walk and we did not have the work permission, our church people they were very kind and were helping us. I did not have apply for NASS that time. And we did not have any funds. Only church people was supporting us.’* (KII 6)
Q4	*‘The only place here I can feel comfortable and friendly is MRANG so every week I'm running here with great pleasure.’ *(KII 1)
Q5	*‘Most time we go to MRANG I go for this erm what's it called, this Monday meeting I meet a lot of people, it's really nice.’* (KII 3)
Q6	*‘If I have any letter from GP, from hospitals, or from any place, and I cannot understand that, I take this letter with me and I come on Monday or Tuesday and can ask anyone from MRANG, to help me, what can I do? Everybody helped me a lot.{That great} And lot of time, they take me to hospitals, go with me to GPs, for I can understand English a little, lot of word I cannot understand. And doctors speak with a big word, medical word but she can understand, told the doctor, and talk it with me, with a small word or what can I understand.’* (FGD 4)
Q7	*‘When (MRANG volunteers) take me to the GP and take me to the Women’s Hospital, I am feeling in my pregnant, I am safe’.* (KII 9)

## Discussion

### Statement of principal findings

The asylum-seeking process has a negative impact on women contributing to feelings of anxiety and/or depression with little control or choice over their future. Women reported feeling stressed regarding poor standard of accommodation, low income, dispersal and the uncertainty of their asylum application outcome. Wellbeing during pregnancy and after childbirth was understood to be multifactorial and women understood that their physical health needs were interlinked and negatively impacted by complex psychological and social factors. Many women reported that although their expectations of maternity services in the UK were often exceeded, key areas such as appropriate information giving, and the use of language interpreters needed to be improved to increase engagement and understanding of their healthcare experiences. Women reported that wellbeing during pregnancy and after childbirth influenced their ability to take care of themselves and their newborn baby and explained that they needed more psychological and social support.

### Strengths and limitations of the study

To the best of our knowledge, this is the first study to explore the understanding and experiences of wellbeing of women seeking asylum (as a distinct group different to women with refugee status and immigrant women) during pregnancy and after childbirth in UK. This study highlights barriers as well as solutions to inform healthcare services that seek to introduce and establish support for women seeking asylum during pregnancy and after childbirth. Practical recommendations were provided from women from seven different countries with varied experiences. All women interviewed recognised that comprehensive maternal health needs (multimorbidity) is an important issue and reported the need for change in support from key services and maternity practice to better improve maternal physical health and psychological and social wellbeing. This study population comprised of women seeking asylum and who attended a supportive charitable organisation in Liverpool and the findings cannot be assumed to be the same in other settings. This study population comprised of women who spoke conversational English and the findings cannot be assumed to be the same for women who do not speak English. However, it may be assumed that maternal multimorbidity in women who do not speak English or who did not engage with charitable organizations are similar or worse because of difficulty accessing care and more complex language barriers [13, 26]. Partners or husbands of women could have been included in the interview process to enable further triangulation of the data and broadened the scope of the topic.

### Relation to other literature

The findings from this study are in keeping with prior research that report multiple barriers to service interaction during pregnancy and after childbirth for immigrant women (including women seeking asylum and who have refugee status). Factors such as language proficiency, unfamiliarity of health services, and poor social determinants, are some of the most common barriers to immigrant women receiving high quality, woman centered maternity care and the information they require to achieve good physical, psychological and social wellbeing during pregnancy and childbirth [4, 5, 13, 26, 27, 39].

Communication between healthcare providers and women who do not speak the language of the host country is one of the most important aspects of providing health information, including how to access services. However, many studies including a systematic review of 29 systematic reviews, report that a lack of interpretation services and lack of access to improve language proficiency leads to inability to express needs adequately and misinterpretation by health providers, hindering the quality of care they receive [26, 39–42].

Moreover, cultural sensitivity goes hand in hand with effective communication and studies have shown that difficulty understanding the needs of an individual can stem from a lack of cultural understanding. For example, in one study included in a systematic review of health-seeking behaviours of immigrants, asylum-seekers and refugees in Europe, exploration of mental health problems was seen to be impeded by the uncertainty of whether their questions had been accurately translated or because words such as ‘depression’ may not be understood in the same way in their native language [43].

Similar to this study, other studies have reported that the housing and material support asylum seekers receive is often inappropriate to their needs, causing a negative impact on their quality of life and physical health as they are unable to afford basic necessities [12, 13, 39, 40, 44, 45]. Financial deprivation has been found to have a direct impact on attending hospital appointments due to limited transport and childcare options [4, 12, 45]. In one intersectional analysis of the causes and experiences of poverty among people seeking asylum and who had refugee status, deprivation was one factor in increasing dependency, disempowerment and lack of agency, also seen in our study, which exacerbated psychological health symptoms [45]. This study also discussed that research into destitution faced by asylum seekers revealed shocking examples of hunger, which was voiced by a few of the women included in this study.

In this study, feeling alone and unsupported was frequently described as one the hardest challenges of being a woman seeking asylum during pregnancy and/or after childbirth, adversely impacting wellbeing by precipitating and/or exacerbating feelings of stress, anxiety, and depression. Loneliness and isolation are commonly reported by people seeking asylum in the UK, resulting in a severely negative impact on their mental health [12, 27, 32, 46]. One study stated that rates of post-natal depression in women seeking asylum may be three times higher than those of women from the UK, owing to stressful life events, lack of social support and cultural factors [44].

Similar to this study, it is well reported that dispersal has a detrimental impact on women seeking asylum during pregnancy and after childbirth as this sudden change of location disrupts essential support systems exacerbating feelings of isolation [12, 13, 26, 47]. Dispersal has been found to directly impact time to initial booking appointment and disrupts continuity of care throughout pregnancy and after childbirth, leading to delayed management of underlying health conditions. In one study, only four of 20 women (who were seeking asylum at the time of interview or had previously sought asylum) interviewed received undisrupted antenatal care and only 30% had first contact with a midwife before 12 weeks gestation which does not meet national guidelines [12]. In accordance with this study, dispersal prevents access to maternity care and breaks down necessary support systems needed in an especially challenging time, exacerbating feelings of isolation and lack of support.

### Recommendations for change in practice

We recommend that healthcare providers should ask all women seeking asylum during pregnancy and after childbirth about their specific healthcare needs and priorities to offer culturally sensitive and non-judgemental support. In keeping with national recommendations, we support the recommendations that equality, diversity and inclusivity training is mandatory and undertaken by healthcare providers to increase their understanding and confidence in responding to social and cultural issues of vulnerable women groups, such as women seeking asylum [48]. Healthcare providers should provide clear information using interpreters at each contact, with more time given to appointments to facilitate effective communication alongside information leaflets in the appropriate language. The development of clinics in hospitals and in community settings that specifically address issues of language and challenges to service access brought about by seeking asylum should be supported [43]. Moreover, provision for someone to interpret during labour and delivery would be beneficial to reduce the risk of traumatic experiences and options should include audio by telephone, video links or face-to-face interpretation services.

Screening for complex health and social needs, particularly mental ill-health should be undertaken at all contacts to offer timely provision of additional support opportunistically. For women without a companion, early assignment of a doula gives women access to non-clinical support that alleviates isolation, improves signposting to maternity services and has been found to reduce adverse birth outcomes in socially disadvantaged women [49].

It would be beneficial for healthcare providers who look after women seeking asylum to understand dispersal policies to mitigate its damaging influence on accessing healthcare services. If a woman seeking asylum is dispersed and needs to change site of care during pregnancy and childbirth, women should be given a handheld printed copy of their medical records that they can take with them to help transfer information about their care.

## Conclusion

Women seeking asylum during pregnancy and after childbirth have complex physical, psychological and social health needs. The process of seeking asylum is challenging as it impacts negatively on women with regards to their choices and independence, resulting in psychological ill-health (stress, anxiety, depression). Women feel particularly stressed regarding dispersal, poor standard of accommodation and the uncertainty of where they would live long-term because of the outcome of their asylum claim. Routine screening, early detection, and management of all health needs along the framework of maternal multimorbidity, during pregnancy and after childbirth must be implemented to better provide woman-centred care [1–3]. This would help to reduce the burden of maternal and newborn morbidity and mortality for women seeking asylum and to improve patient safety, experience and wellbeing. A multidisciplinary team, with links and effective referral pathways to maternal mental health and social services, are necessary for women seeking asylum, to ensure a more integrated, comprehensive assessment of maternal multimorbidity and to provide maternity care in a way that meets all health needs. Although the women included in this study shared negative experiences despite receiving support from a charitable organisation, this study shows that the support they received was invaluable and therefore ways to include working alongside charitable organisations within maternity care should also be sought.

### Supplementary Information

**Additional file 1: Supplementary file 1.** In-depth interview and FGD topic guides.

## Data Availability

The dataset used and analysed during the current study are available from the corresponding author on reasonable request.

## Abbreviations

FGD: Focus group discussion
KII: Key informant interview
MMBRACE-UK: Mothers and Babies: Reducing risk through audits and confidential enquiries across the UK
MRANG: Merseyside Refugee & Asylum seekers Pre and Post Natal Support Group
NASS: National Asylum Support Service
NHS: National Health System
NIC: National Institute for Health Care Excellence
PTSD: Post-traumatic stress disorder
RWC: Refugee Women Connect
