# Supplementary material for: Maternal multimorbidity - experiences of women seeking asylum during pregnancy and after childbirth: a qualitative study
Source: BMC Pregnancy Childbirth. 2023 Nov 13;23:789. doi: 10.1186/s12884-023-06054-x (PMC10641960; doi:10.1186/s12884-023-06054-x)
Supplement: Supplementary file 1 — Additional file 1: Supplementary file 1. In-depth interview and FGD topic guides. [file 12884_2023_6054_MOESM1_ESM.docx]

**Supplementary file 1: In-depth interview and FGD topic guides**

maternal multi-morbidity - experiences of women seeking asylum during pregnancy and after childbirth: a qualitative study

**Introduction:** Thank the participant for agreeing to take part in the study and for allowing the interview to be recorded. Check with participant that they still want to continue and if so, gain verbal consent on the recording. Ask the participant if they have any questions before starting.

In-depth interview

Part one (open, allow the participant to tell their story)

**Objective 1: To elucidate the impact of the asylum-seeking process on the perinatal wellbeing of asylum-seeking women**

1. Participant’s experiences of being pregnant and giving birth as an asylum seeker

*Prompts:*

*-Positive/negative experiences*

*-Support system- how has that changed?*

*-Psychological/physical/social implications of being pregnant and giving birth as an asylum seeker*

**Part two** **(more structured)**

**Objective 2: To identify the different ways that refugee and asylum-seeking women understand perinatal wellbeing**

1. Meaning of perinatal wellbeing to the participant (might need an explanation of terms- perinatal: the period from week 22 of pregnancy till one completed week after birth)

*Prompts:*

*-What makes you feel well during pregnancy?*

*-What makes you not feel well during pregnancy?*

*-What helped you when you gave birth? (if relevant)*

*-What did you find unhelpful when you gave birth? (if relevant)*

*-What helped you feel well after you had given birth?*

*-What is a good pregnancy to you?*

*-What is a good birth to you?*

1. The participant’s perception on what is the most important factor to promote perinatal wellbeing

*Prompts:*

-*Social/physical/psychological factors*

1. The participant’s experiences of perinatal services in the UK

*Prompts:*

*-Positive/negative experiences*

*-In what way do/don’t they meet the participant’s needs?*

**Objective 3: To examine the sociocultural challenges faced by perinatal asylum-seeking women living in the UK**

1. The sociocultural challenges the participant faces in the UK

*Prompts:*

*-Social networks*

*-Basic living (buying essentials, clothing, where you live)*

*-Stigma/discrimination*

*-Language*

*-Religion*

1. The impact of sociocultural challenges on the participant’s perinatal wellbeing

*Prompts:*

*-Positive/negative impact*

1. The differences in perinatal wellbeing between UK and the participant’s home country (if relevant)

*Prompts:*

-*positive/negative*

*-Support system better/worse*

*-healthcare better/worse*

**Ending the interview:**

Is there anything the participant would like to add?

What would you like to come from this study?

Thank the participant.

**Focus Group Topic Guide**

General experience-> specifics

9 Areas

1. Access to maternity care
2. Needs
3. Health professionals
4. Information giving
5. Appointments
6. Extra support and Language
7. NHS system
8. Culture
9. Service improvement

**Start:**

- Confidentiality
- Respect each other talking and differences of opinion (we all have different backgrounds and experiences)
- Explain the aim of today- exploration of hopes and expectations of maternity care in UK

**General experiences:**

When you became pregnant/found out you were pregnant in the UK, what maternity service did you hope for? Expect? (If different, how and why did you have this expectation?)

What was your first experience of maternity care in the UK? –How different was this to home?

What do you find good/bad about maternity services? Why?

**Specifics:**

1. **Access**

How did you find out how to receive maternity care in the UK? Was it easy/not to find out and access maternity care?

Who was involved in telling you? Did they do this well?

Did your access to maternity services change at any point? Why? What impact did this have?

1. **Needs**

What health needs do you have?

Do you feel that your needs as a pregnant woman were met by the maternity services?

What needs are particularly important to you and you expect to be addressed?

1. **Health professionals**

What have your interactions with health professionals been like?

What you hoped for/expected? If so/not-why?

Do they make you feel comfortable and confident in your care?

Do they meet your needs? (Try and echo what they said their needs were at this point if relevant)

1. **Information giving**

Do you feel you have been given enough information to understand maternity care and your own pregnancy and birth?

Has information been given to you in a helpful and understandable way?

1. **Appointments**

Have appointments been appropriate for you? (E.g. length/amount/quality/regularity)

Have you been able to attend all your appointments? If so/not why?

Was this what you hoped for/expected and why?

1. **Extra support and language**

Have you received any extra support? (Antenatal classes/support groups/language aids) If so/not     why? Have these been helpful?

1. **NHS system**

Do you feel you understand the NHS system and what to do when you are worried about your pregnancy or birth?

Do you feel confident in the NHS to promote your perinatal wellbeing?

1. **Culture**

Have your own cultural practices been incorporated into your pregnancy care? Is this important to you?

1. **Service improvement (hopes and expectations for future care)**

As an asylum seeker, what do you think would make your perinatal experience better here in the UK?

-Needs/health professionals…

**FINAL QUESTION if time allows:**

Do you feel your experiences of NHS maternity care have matched your hopes and expectations?

If so/not why? How has this impacted your pregnancy and birth?

END

- Ask if anyone has anything to add
- Summarise and check for correct meaning
- Thank and finish
